# Pharmaceutical marketing: the example of drug samples

**DOI:** 10.1186/s40545-022-00479-z

**Published:** 2022-11-07

**Authors:** Emily Couvillon Alagha, Adriane Fugh-Berman

**Affiliations:** 1 Dahlgren Memorial Library, Georgetown University Medical Center, 3910 Reservoir Rd NW, Washington, DC 20007 USA; 2 Department of Pharmacology and Physiology, and Department of Family Medicine, Georgetown University Medical Center, 3900 Reservoir Rd NW, Med-Dent SE402, Washington, DC 20007 USA

**Keywords:** Drug samples, Pharmaceutical marketing, Branded drugs, Gifts, Adherence

## Abstract

Branded drug samples are one of the most important promotional tools that pharmaceutical manufactures employ. Pharmaceutical sales representatives (“drug reps”) use samples to gain access to physicians and other prescribers. Sample provision is closely intertwined with visits by drug reps; detailing visits convince physicians to try new products, while sampling maintains the flow of prescriptions. Only drugs with the highest profit margins are sampled. Although physicians believe that samples save patients money, patients who receive samples have higher overall out-of-pocket costs. Most studies have found that patients in financial need are least likely to receive samples. Pharmaceutical marketers pitch samples as a low-risk way to deal with diagnostic uncertainty. In fact, there is no evidence that samples aid diagnosis. Sample availability may compromise patient safety by reducing compliance with guidelines and steering patients towards newer drugs, for which adverse effects have not been well-delineated. Although physicians believe that samples improve adherence for low-income patients, branded samples do not improve access or adherence Samples are not a charitable activity, but are instead a highly effective form of drug marketing. Sampling of branded drugs increase drug costs for everyone. Only a cohesive effort by clinicians, legislators and policymakers can end this practice. Evidence supports a ban on sample distribution of branded products.

## Introduction

Physicians’ use of prescription drug samples has been debated. Some argue that samples decrease prescribing quality and increase overall prescription drug costs [1–3]; while others argue that free drug samples are beneficial for patients [4, 5]. In fact, drug samples are a powerful marketing tool. In pharmaceutical marketing literature, samples are described as “one of the most important promotion instruments” [6] and the “soul of selling in the prescription drug industry” [7]. This paper will discuss the purpose and effect of sampling, and how samples are viewed by physicians and by industry. Additionally, we will make recommendations for restricting or banning sampling.

In 2016, pharmaceutical companies invested $13.5 billion in sample distribution in the United States [8]. A survey of 3167 U.S. physicians in 2003–2004 found that 78% accept drug samples [9]. A 2018 survey of 33 family medicine teaching units in Quebec found that most physicians accepted samples [10]. Sample use varies across specialties and indications; for example, the use of free samples is higher in dermatology compared to other specialties [11]. Little research on sampling appears in the medical literature; a comprehensive survey of literature from 1986 to 2002 identified 23 research papers. Most papers focused on physician attitudes towards sampling, and the majority of those studies found that physicians viewed samples favorably [12].

Sampling is a well-established marketing strategy in many consumer-facing industries, including food and beverages, luxury cosmetics, and print media [13–15]. Samples appeals to both informational and affective needs in customers, and increase the probability that the customers will choose the sampled brand in the future [16, 17]. One study of six sales data sets found that providing a wide variety of new product samples in stores  increased sales immediately, an effect that persisted 2–8 weeks later [18]. Sampling is more effective than coupons or other marketing tactics and produces sustained changes in purchasing habits that can last up to 12 months [19].

Although prescription drugs are paid for and consumed by patients, the intermediary role of the physician means that “the chooser is not the user” [20]. Pharmaceutical sampling targets prescribers because from the manufacturer’s perspective, physicians are the customers. The intermediary role played by physicians mirrors other business-to-business (B2B) relationships. B2B marketing strategies focus on building sustained personal relationships with gatekeepers and decision-makers within a supply chain who influence how the product reaches individual consumers [21]. In an era of increasing regulations on physician–pharma interactions, samples play a crucial role in maintaining these gatekeeper relationships.

Sample provision is closely intertwined with visits by drug reps. Physicians value samples: one study found that 84% of physicians considered drug samples to be the most important service provided by drug reps [22]. Sample drops are used as “physician access enablers”, and without samples, “many detailing encounters with physicians may not happen” [6]. Drug reps bring small quantities in order to have a reason to visit physicians every other week or so.

Detailing visits are an acquisition tool to convince physicians to try new products, and sampling maintains the flow of prescriptions [23]. When physicians meet with pharmaceutical representatives, they increase the number of samples they dispense [24]. Marketing literature advocates sampling only products that meet a “minimum markup threshold”, meaning drugs with the highest profit margins [25]. Pharmaceutical companies optimize profitability by tracking the use of samples to ensure that they are not being given out so generously that sales are cannibalized rather than enhanced [6, 26]. Sample drops, in conjunction with detailing, have the highest return on investment (ROI) among marketing tactics [27].

## Why physicians like samples

Why do physicians think it is beneficial to accept samples? Physician surveys have identified several motivating factors, including reducing costs for patients, evaluating treatment efficacy, demonstrating proper use, starting therapy promptly, increasing patient convenience and satisfaction, improving patient compliance, and treating short-term medical problems [28, 29]. Sample closets often serve as communal medicine cabinets, supplying physicians, office staff, friends, and family [25, 30, 31].

Samples are dispensed most often to patients who are newly diagnosed, or were previously diagnosed but were prescribed a different drug on a previous visit [19]. Pharmaceutical marketers pitch samples as a low-risk way to “find the best patient–drug match” [7]. A marketing research article states that physicians’ “greater diagnostic uncertainty … induces their increased prescriptions of the drugs with samples” [7]. Early-career physicians are a particular target, as well as physicians working with high-uncertainty disease categories, including asthma and allergies [7]. Among new doctors, samples increase the likelihood of a prescription by 81%, compared with a 51% uptake rate among doctors who received a detail-only visit without samples [32].

Pharmaceutical representatives provide information, flattery, and samples to persuade clinicians that they are making wise therapeutic choices [26]. Samples may be a seductively simple way to deal with diagnostic uncertainty; they can decrease physician anxiety and increase dependence on sample drops. In fact, there is no evidence that samples aid diagnosis.

Samples are not cost-effective, either. Although physicians (and some patients) believe that samples save patients money [33], samples do not provide long-term financial benefits. Patients who receive samples have higher overall out-of-pocket costs [34]. Also, most studies have found that patients in financial need are least likely to receive samples. In one analysis of patients over 65 with government-funded insurance in the United States, higher-income patients were more likely than low-income patients to receive samples over a year [35]. A survey of 32,681 patients also found that samples predominantly go to wealthier, insured patients [36]. A survey of 200 patients with asthma in Chicago found that only 4% of those on public aid received samples, compared to 20% of uninsured, “self-pay” patients and 31% of insured patients [37]. The strongest predictor of receiving samples is the number of office visits, not financial need [38].

## Samples influence prescribing

Samples habituate physicians to prescribe specific drugs. An analysis of physician prescription decisions found that samples positively influenced prescribing decisions in two ways: by increasing base prescription rates and enhancing physician susceptibility to detailing visits [39]. Subsequent studies found that sample availability positively influences physician adoption of targeted drugs [40, 41]. One modeling study inferred that sampling was effective in physicians who saw patients with private insurance but not for those who saw patients with Medicare (government-funded health insurance for elders in the U.S.) or who were in a health maintenance organization (HMO; a type of health insurance where patients pay a set fee for a range of provided services) [20].

The availability of samples can lead physicians to prescribe drugs that differ from their preferred treatment. A cross-sectional U.S. survey asked 154 family medicine and general physicians to select treatments for patients with urinary tract infection, hypertension, or depression. Each hypothetical scenario was accompanied by a list of available samples. Among participants who dispensed samples, 49% to 95% (depending on scenario) were willing to dispense a sample that differed from their preferred drug choice [28]. Eighteen years later, Quebec studies found similar results: half (51%) of health care providers provided the patients with a drug sample even if it was not their first choice for treatment [10].

A systematic review of 19 studies of interactions between practicing physicians and pharmaceutical companies found that lower physician prescribing quality was associated with industry interactions, including the acceptance of free drug samples [42]. Acceptance of samples begins early in training [11]. Premedical students are exposed to sampling activities while participating in volunteer activities. A survey of 911 pre-matriculated medical students found that 34% observed their supervising clinicians receiving samples, and 7% reported receiving samples themselves [43].

Sample closets in residencies counter evidence-based prescribing. A randomized trial of 29 internal medicine residents found that residents with access to sample closets were less likely to prescribe unadvertised drugs and over-the-counter drugs than residents without sample access, since a generic alternative was available [44]. Boltri et al. found that both residents and attendings were less likely to prescribe first-line anti-hypertensive drugs when samples of second-line treatments were available [45]. In Vermont, primary care physicians who had sample closets were less likely to prescribe the preferred antihypertensive according to current guidelines [46]. A study of family medicine clinics found that physicians at a clinic that allowed samples were much more likely to prescribe sampled medications (than at two similar clinics that did not allow samples [47]).

Gifts to physicians have been shown to bias prescribing, but many physicians do not consider samples to be gifts [1, 48, 49]. Physicians seem to view the practice in isolation from other detailing efforts, although samples, like all gifts, beget social expectations.

## The risks of samples

Besides influencing prescribing patterns, sample availability may compromise patient safety by reducing compliance with guidelines and steering patients towards newer drugs, for which adverse effects have not been well-delineated. Adverse effects from samples are not tracked consistently with regular pharmacovigilance data [50]. Sample provision may not even be documented, complicating adverse event reporting; only two-thirds of clinicians in the Quebec study (64%) recorded providing samples in patient records [10]. Although physicians believe that samples improve adherence for low-income patients, branded samples do not improve access or adherence. Samples are usually for expensive, chronically used drugs, and may drive up overall costs [51]. For example, sample use has increased among insulin users in recent years, and is associated with higher per-prescription costs over nonusers of samples [52]. Samples may have increased prescription rates for expensive insulin delivery systems that provide little or no benefit in patient outcomes.

Samples can lead to discontinuity in treatment after patients run out of samples of a drug they cannot afford. Receiving 30-day samples of generic drugs, on the other hand, increases adherence [53]. Adherence to generic drugs is higher than to branded drugs, probably because patients can afford them [54]. A pilot project by a managed care organization found that physicians prescribe generic drugs more when the sample closet is filled with generics [55].

Many practices fail to store sample drugs safely. Some hospitals have allowed pharmaceutical representatives to stock and monitor sample closets [56]. A 2005 study of 31 primary care offices found that medications with different routes of administration were stored together (considered an unsafe practice) in 81% of offices [56]. Less than 15% of the offices separated look- or sound-alike and/or similar packaging products from other products in the sample inventory area. In the U.S., physicians carry legal liability for the risks that samples pose to patients, even when patient harm is caused by inadequate labeling of sample packaging [57]. There are environmental concerns as well. Waste generated from drug samples is estimated to be 5740 metric tons per year [58].

Sampling is banned in many academic institutions, including the University of Michigan health system and Stanford University Medical School [59]. Some physician practices refuse samples as well [60, 61].

However, physician dispensing of drug samples is still considered acceptable by the American Medical Association and the American Academy of Family Practitioners [62]. Additionally, the FDA temporarily loosened restrictions on sampling in response to the COVID-19 pandemic [63]. Because many offices have reduced in-person interactions, including drug rep and patient visits, samples can now be shipped directly to patients. This new guidance makes it easier for pharmaceutical companies to distribute samples in spite of decades of evidence that samples cause harm. With little guidance at the national level, the decision of whether or not to accept samples in private practice is largely left to individual physicians.

## Recommendations and conclusions

Samples are not a charitable activity, but are instead a highly effective form of drug marketing. Samples remain the largest marketing investment among most companies. Pharmaceutical companies would not invest so much in optimizing sampling distribution if they did not see a return on investment from these strategies.

The sampling of branded drugs increases drug costs for everyone. While some countries, states and other jurisdictions have laws and regulations that address gifts to physicians, drug sample provision is always excluded. This needs to change: samples are gifts. In the meantime, individual prescribers have the power to change this practice by refusing samples. Only a cohesive effort by clinicians, legislators and policymakers can end this practice. Laws that address gifts to prescribers should always include samples as gifts. Evidence supports a ban on sample distribution of branded products.

## Data Availability

Not applicable.

## Abbreviations

B2B: Business-to-business
FDA: The Food and Drug Administration
ROI: Return on investment
HMO: Health Maintenance Organization
